# Two-step machine learning method for the rapid analysis of microvascular flow in intravital video microscopy

**DOI:** 10.1038/s41598-021-89469-w

**Published:** 2021-05-11

**Authors:** Ossama Mahmoud, Mahmoud El-Sakka, Barry G. H. Janssen

**Affiliations:** 1 Department of Computer Sciences, Western University, London, ON N6A 3K7 Canada; 2 Department of Medical Biophysics, Western University, London, ON N6A 3K7 Canada; 3 Centre for Critical Illness Research (CCIR), Lawson Health Research Institute, London, ON N6C 6B5 Canada; 4 Kidney Clinical Research Unit (KCRU), Lawson Health Research Institute, London, ON N6C 6B5 Canada

**Keywords:** Image processing, Circulation, Blood flow, Computational biology and bioinformatics, Physiology

## Abstract

Microvascular blood flow is crucial for tissue and organ function and is often severely affected by diseases. Therefore, investigating the microvasculature under different pathological circumstances is essential to understand the role of the microcirculation in health and sickness. Microvascular blood flow is generally investigated with Intravital Video Microscopy (IVM), and the captured images are stored on a computer for later off-line analysis. The analysis of these images is a manual and challenging process, evaluating experiments very time consuming and susceptible to human error. Since more advanced digital cameras are used in IVM, the experimental data volume will also increase significantly. This study presents a new two-step image processing algorithm that uses a trained Convolutional Neural Network (CNN) to functionally analyze IVM microscopic images without the need for manual analysis. While the first step uses a modified vessel segmentation algorithm to extract the location of vessel-like structures, the second step uses a 3D-CNN to assess whether the vessel-like structures have blood flowing in it or not. We demonstrate that our two-step algorithm can efficiently analyze IVM image data with high accuracy (83%). To our knowledge, this is the first application of machine learning for the functional analysis of microvascular blood flow in vivo.

## Introduction

Intravital video microscopy (IVM) allows investigating the blood flow in small blood vessels in living tissue in great detail. Intravital microscopic brightfield observations can generally be performed in any tissue that can be accessed surgically, exteriorized for visual observation, and is transparent enough to allow visualization of the microvasculature. As such, IVM has been used to visualize the microcirculation in a wide range of tissues and animal models^1^. It has been used for the in-vivo observation of the flow of red blood cells (RBC) and their role in oxygen transport^2^, the adhesive behavior of leukocytes and their role in immune responses^3^, and the behavior of platelets and their role in the thrombus formation during blood clotting^4^. Moreover, IVM has proven to be a valuable tool for the investigation of vessel growth^5^, tumor growth, and metastasis^6^, as well as the pathophysiology of the microvasculature during different diseases in different tissues and organs^7–9^.

IVM is used for the in-vivo investigation of the muscle microvasculature during sepsis. This condition involves a severe and uncontrollable inflammatory response associated with pathophysiological changes distant from the inflammation's primary site. It is one of the major causes of patient mortality in intensive care units^10^. Research shows that microvascular blood flow remote from the initial infection becomes disturbed during sepsis, leaving large parts of microvasculature under-perfused and functionally compromised^11–15^. Moreover, these perfusion disturbances develop within several hours after inducing sepsis^16^, indicating that Microvascular Dysfunction (MD) is associated with the inflammatory process's early stages. Since such a disruption of blood flow directly impedes tissue metabolism, organ and tissue function are directly affected. Indeed, organ dysfunction in sepsis is directly related to the severity of the septic inflammatory response syndrome^10^.

IVM has proven to be a valuable tool for investigating microvascular blood flow under different physiological conditions. By analyzing the digital microscopic images, it is possible to investigate and quantify tissue perfusion during sepsis by determining the number of perfused capillaries and quality of blood flow of the observed tissue^17^. Digital cameras have significantly improved over the last decade, resulting in higher acquisition speeds and better image quality. However, the ultimate analysis of the experimental video data still requires visual inspection of the image data, which is time-consuming and impedes an efficient investigation of the acquired IVM video data. Moreover, to ensure the visual analysis's accuracy, two or more independent analyzers often perform the analysis, increasing this problem even further.

The leading cause for the off-line analysis's time-intensive character is the complexity of the video images. Usually, these contain many almost identical stagnant or dynamically moving structures (i.e., red blood cells), as well as sudden changes in contrast or movement artifacts of the tissue. Therefore, the image analysis requires a visual assessment of all the recorded videos, sometimes on an image-to-image basis. Moreover, the experiments performed in this paper were recorded using a newly designed multispectral camera system containing four integrated CMOS camera units (paper in progress). These cameras can acquire digital images at video framerates up to 30 to 40 frames per second, resulting in image data volumes exceeding 100 Gb per experiment. Clearly, the image dataset's manageability becomes very challenging at these volumes, again underpinning the need for a computer-assisted analysis method.

In recent years the automated analysis of the microcirculation has focussed primarily on blood vessel segmentation and is often used for diagnostic purposes in laryngology, oncology, ophthalmology^18^. Vascular segmentation mainly focuses on the automated analysis of the vessel structure layout and relies on the light-absorbing properties of RBC in the microvasculature and is commonly used to analyze the structural layout of the observed microcirculation. As such, many algorithms exist to segment vessel structures. The most preliminary and basic algorithm is the Steger Unbiased Detector of Curvilinear Structures (SUDCS), which relies on measuring gradients across the image and uses these values to model a mathematical structure of a curved line^19^. Over the years, the algorithm has gone through several improvement steps^20–22^, and it is still considered a robust vessel segmentation algorithm.

However, despite all these improvements, the SUDCS algorithm does not take blood perfusion in the observed vessel segments into account. Since the functionality of microcirculatory perfusion is a critical parameter for the assessment of MD, it is not only important to assess the structural layout of the observed vasculature, but it also requires evaluating microvascular perfusion. Recently, two studies proposed image processing algorithms for the automated detection of blood flow in microvessels. While the Temporal Sidestream Dark Field Image Contrast Analysis (tSICA) algorithm examines the changes in intensity associated with flowing blood^20^, the Epipolar-Plane Image Analysis (EPIA) algorithm determines blood flow by a one-directional projection of the vessel structure directly from the intravital microscopic videos^23^. However, both algorithms showed limited applicability when processing images with higher blood flow velocities.

Microvascular perfusion is a highly dynamic process in living tissue. It can range from vessel segments with either no flow, only a few flowing RBC, to entirely filled with stagnant RBC, or well perfused with high-velocity blood flow. Since microcirculatory perfusion is a critical parameter for assessing microvascular function, it is not only essential to evaluate the structural layout of the visible vasculature but also requires the evaluation of its perfusion. However, so far, no automated processes are availably capable of assessing both parameters automatically simultaneously. In this paper, we present a two-step machine-learning-based algorithm for the functional and structural analysis of microcirculation. The first step of the algorithm uses an adaptation of the well-established (SUDCS) to segment the vessel structures^19^. While the second uses a specifically designed and trained 3D Convoluted Neural Network (3D-CNN)^24^ to determine whether a vessel segment carries blood flow or not. To our knowledge, there has been no machine-learning-based approach to the functional analysis of microcirculation.

## Results

### Performance of the 3D-CNN

For this study, we used an image data set of in total 458 IVM videos divided into two separate sets. One set (392 videos containing 27,809 data points; 20,382 positive and 7427 negative samples) was used for 3D-CNN training only, while a different collection of 66 videos (4404 labeled data points; 3362 positive and 1042 negative samples) which had not been used for any previous training, was used for testing.

Tables 1 and 2 summarize the performance of the 3D-CNN. Table 1 shows the confusion matrix, which summarizes the performance of the 3D-CNN model to classify the identified vessel segments as flowing or non-flowing correctly. It summarizes the distribution of errors and predictions made by the model, i.e., the number of times a vessel segment is correctly identified as flowing (True Positives; TP = 3179) or incorrectly identified as non-flowing (False Negatives; FN = 183), or the number of times a non-flowing vessel is correctly identified as non-flowing (True Negatives; TN = 787), or incorrectly identified as flowing (False Positives; FP = 255). For the simple Logistic Regression, these numbers were 742 (TN), 556 (FN), 2806 (TP), and 300 (FP), respectively.

**Table 1:** Confusion matrix of 3D-CNN on test set: accuracy of prediction (n = 4404 data points).

	Predicted not flowing	Predicted flowing
**3D-CNN**		
True not flowing	787 (TN)	255 (FP)
True flowing	183 (FN)	3179 (TP)
**Simple logistic regression**		
True not flowing	742 (TN)	300 (FP)
True flowing	556 (FN)	2806 (TP)

*TN *true negative,* FP *false positive*, FN *false negative*, TP *true positive.

**Table 2:** Derivations of the confusion matrix.

Evaluation metrics	Accuracy	Positive precision	Sensitivity	f1-score	Negative precision
3D-CNN	0.90	0.93	0.95	0.94	0.81
Logistic regression	0.81	0.90	0.83	0.87	0.57
Equation used	$\frac{TP+TN}{TP+TN+FN+FP}$	$\frac{TP}{TP+FP}$	$\frac{TP}{TP+FN}$	$\frac{2TP}{2 TP+FP+FN}$	$\frac{TN}{TN+FN}$

*TN *true negative,* FP *false positive*, FN *false negative*, TP *true positive.

The actual accuracy or percentage of points correctly identified by the 3D-CNN algorithm or a simple logistic regression model and the equations used to calculate the metrics are presented in Table 2. Analysis of the 3D-CNN performance on the 4404 data points shows that the network has an overall accuracy of 90% in determining whether blood flow in a vessel segment is flowing or not. The sensitivity/recall of the model is 95% indicating that the CNN can identify true positives well. The positive predictive value is 0.93, showing that the CNN correctly predicts a flowing vessel to be flowing in 93% of the cases. Finally, the F1-score is 0.94, being the harmonic mean of precision and recall, showing that the model has high precision and recall for flowing vessels^25^. Conversely, the negative predicted value is 0.81, indicating that the CNN accurately predicts a vessel to be non-flowing in 81% of the cases. Compared to a baseline assessment using a logistic regression model, the 3D-CNN performed better in accuracy, positive predictive value, f1-score, sensitivity, and negative predictive value.

Our results suggest that the 3D-CNN can efficiently predict and identify which vessel segments in the microscopic Field of View (FOV) during IVM are perfused with blood flow. However, the model appears to have more trouble with non-flowing structures. The accuracy of both the prediction and actual identification of non-flowing vessels is lower than for perfused vessel segments, resulting in a lower true-negative and a higher false-positive rate.

### End-to-end testing

The entire algorithm's ultimate performance was tested by applying it to 34 new IVM videos that had not been manually analyzed or used for training purposes. This test procedure aims to mimic the complete 2 step algorithm's application and eliminate the initial manual analysis procedure.

**Figure 1:**
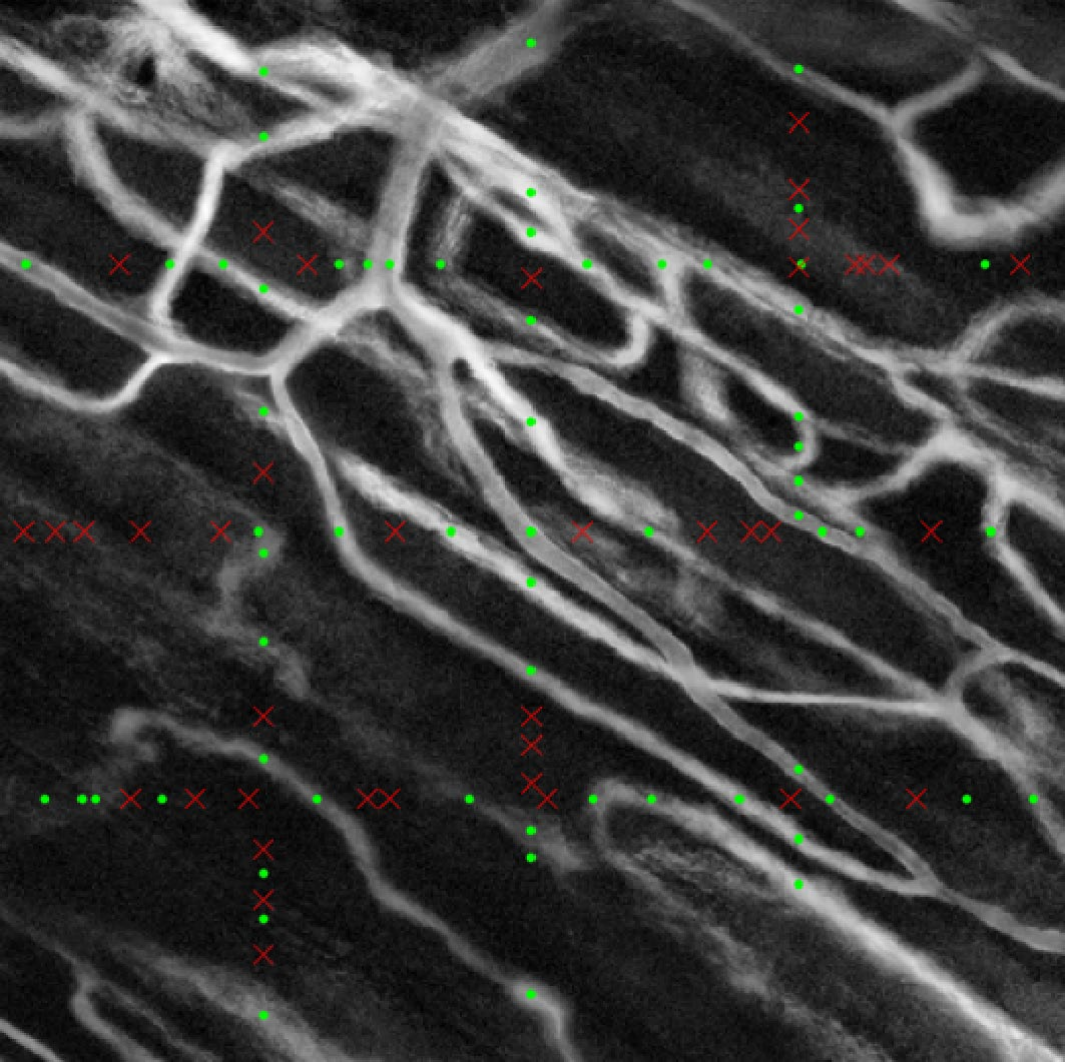
Final assessment image. Image showing for the final assessment of the two-step algorithm combined with a SAD image from an intravital microscopic video. Green markers indicate a perfused microvessel, red markers indicate a non-perfused artifact, discovered by step 1 (SUDCS algorithm), but then subsequently rejected in step 2 by the 3D-CNN^45,46^.

The two-step algorithm's results are then manually reviewed, determining the percentage of missed vessels that are not captured by the algorithm and the error rate caused by misidentifying a non-flowing vessel as flowing. We find that the overall accuracy of the 2-step algorithm is about 83% of flowing vessels detected by a human reviewer, with an error rate of only 3.5%. This result indicates that although the algorithm has difficulty capturing all vessels as identified by subsequent manual analysis, all the identified vessels are accurately labeled by the algorithm as perfused or not. Figure 1 depicts an example of a final manual assessment of the 2-step algorithm described in a SAD image of an intravital microscopic video. The vessel segments intersecting with the equidistant reference lines are marked with 92 green and 20 red markers. Green markers identify correctly identified perfused vessel segments, while red markers signify non-perfused artifacts that were initially positively identified by the SUDCS but were later rejected by the 3D-CNN algorithm. These results indicate that the trained 3D-CNN in the second step of the 2-step algorithm is capable to forcefully reject initially misidentified actively perfused vessel segments directly from the captured IVM videos.

## Discussion

This study shows that a two-step 3D-CNN-based algorithm can be used for the off-line analysis of intravital video microscopic images. These images are generally stored on a hard disk for later manual off-line analysis, which is time-consuming and challenging work. By designing a two-step algorithm, we can now effectively identify vessel segments using a modified SUDCS and determine whether these microvascular parts are actively perfused with blood flow using a trained 3D-CNN. As such, it is now possible to significantly expedite the analysis of the intravital microscopic observation. While manual analysis of one complete experiment can easily take up one to two days depending on the size of the acquired video data, the proposed two-step algorithm can efficiently perform the same task in a matter of minutes.

Microvascular dysfunction involves a rapid decline in local tissue perfusion and can often be observed during severe systemic inflammatory and other pathophysiological conditions. In a severe systemic inflammation like sepsis, it is a commonly occurring critical systemic feature, significantly impeding an adequate flow of blood to tissue and organs, resulting in multiple organ failure and patient mortality. Several pathologies have been associated with MD including, diabetes^26^, severe obstructive sleep apnea^27^, heart failure^28^, autoimmune conditions^29^ as well as neurological pathologies related to acute ischemic stroke and Alzheimer's disease^30,31^, all suggesting that MD constitutes a pathophysiological element found in a range of different conditions. As such, the ability to efficiently quantify microvascular perfusion is of great importance for understanding the pathophysiological role of MD. However, so far, it has mainly been investigated in relation to sepsis or endotoxemia^32^.

Direct experimental visualization of MD's onset and development is usually done by visualizing the microcirculation in surgically exposed tissue of a living animal with IVM. It allows for detailed visualization of all elements of the microvasculature. Microvascular dysfunction can be quantified with intravital microscopy by observing one of the most abundant cellular blood components in the tissue, i.e., RBCs flowing through the microvessels.

As such, all raw experimental IVM images are pre-processed before analysis. During this pre-processing, each pixel's absolute intensity differences between consecutive images during the complete IVM recording are collected to highlight the areas in which RBC are flowing. It visualizes the vessel geometry associated with RBC flow, which can be used for further analysis. This process was further enhanced by removing movement artifacts and applying subsequent Contrast Limited Adaptive Histogram Equalization (CLAHE) filtering, showing that it is possible to improve the visibility of vessel structures in the FOV. By enhancing the vessel layout in the Sum of Absolute Differences (SAD) image, the SUDCS algorithm can more easily perform a segmentation of the vessel layout in the FOV.

**Figure 2:**
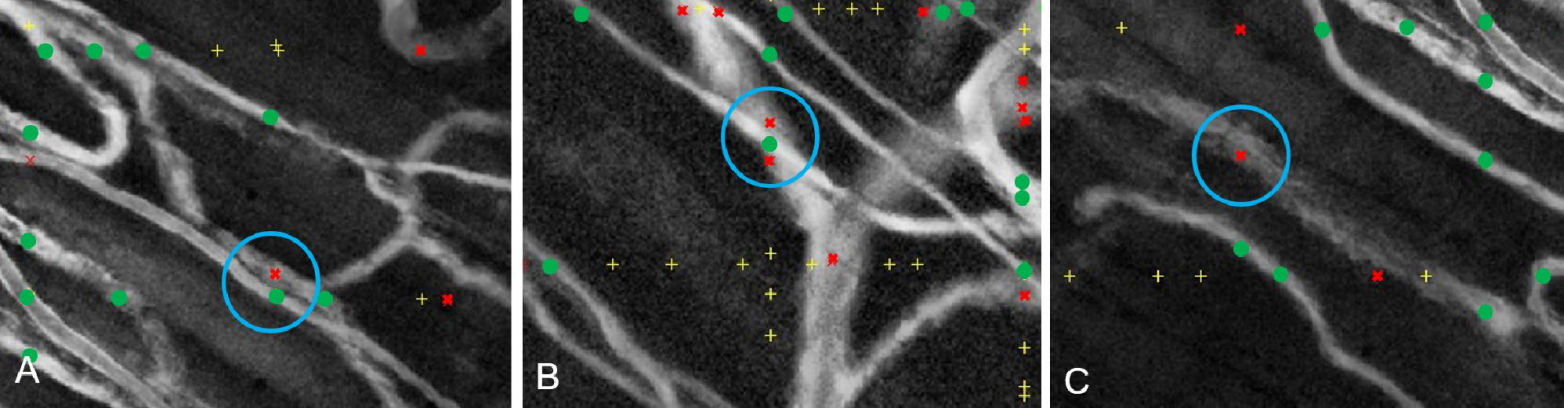
SUDCS Identification. **(A)** Example of three commonly SUDCS vessel identification errors. Green markers: vessel identified by both the SUDCS and manual analysis. Yellow markers: identified by SUDCS. Red markers: identified by manual analysis. **(B)** Two adjacent vessels are shown as one vessel by SUDCS. **(C)** Two vessels intersecting each other are shown as one vessel by SUDCS. Poorly outlined vessel structure due to vessel depth prevents SUDCS from detecting vessel^45,46^.

Our analysis shows that the first (SUDCS) step of the 2-step algorithm appears to be less efficient in correctly capturing the vasculature's geometric outline in the enhanced SAD images. Our study results indicate that although the first step (SUDCS) of the 2-step algorithm can capture almost all true vessel structures in the SAD image, it also falsely labels artifacts in the SAD image as potential vessel segments. Furthermore, our analysis shows that the SUDCS algorithm also appears to have difficulty in correctly capturing closely adjacent or intersecting vessel structures, as well as less defined vessel segments of deeper blood vessels (for example, see Fig. 2).

Despite these limitations, we find that the second step of the algorithm (3D-CNN) effectively removes these initially incorrectly identified vessel structures. Moreover, our results indicate that the trained convolutional network can classify the identified vessel segment intersections as flowing correctly. Conversely, the trained 3D-CNN appears to have more difficulty in identifying non-flowing structures in FOV. It is not entirely clear why the trained convolutional network tends to underestimate the presence of non-flowing structures in the FOV. Although this likely results from the high amount of false positives detected by the algorithm's first step, this inaccuracy could be further aggravated by optical artifacts in the observed tissue. The 3D-CNN algorithm uses variations in light intensity in the area surrounding the identified intersecting points between reference lines and the vessel segments to label a vessel segment as either perfused or not. Since these light changes likely originated from optical tissue artifacts not related to blood flow or from in vessel segments outside the microscopic focal plane, it is conceivable that the algorithm may incorrectly label these artifacts as perfused vessel segments.

Moreover, since our laboratory regularly performs IVM observations to investigate the microvasculature under different pathophysiological conditions, additional experimental training data will become available to further train and improve the 3D-CNN algorithm. Besides, the current algorithm only addresses the question of whether an identified vessel segment carries blood flow involving moving RBCs. It currently provides no information about other important hemodynamic parameters critical for tissue perfusion, i.e., vessel diameter and flow velocity, which would give even more detailed functional information about the observed microvasculature's perfusion.

Our results provide a first example to use AI to analyze the observed microvasculature during IVM. Following the introduction of the SUDCS algorithm^19^, there has been significant improvement in vessel segmentation using blood cell tracking^20,22^, while the SUDCS algorithm's accuracy was further improved by combining the initial algorithm with a pre-processing and enhancement step^20,21^. Moreover, while the tSICA algorithm examines the changes in intensity associated with flowing blood^20^, other investigations show that the presence of blood flow can be determined by a one-directional projection of the vessel structure using an EPIA algorithm directly from the intravital microscopic videos^23^.

Furthermore, in this study, we expanded on this development by improving the vessel segmentation using a morphological close operator^26^ to fill in the binary vessel images, followed by removing all segment structures shorter than 4 pixels.

This study describes an automated analysis method capable of quantifying the functionality of microvascular blood flow directly from the acquired images. Without the need for any manual evaluation, IVM images can now be evaluated using a combination of an improved SUDCS algorithm and 3D-CNN-based machine learning step, achieving an overall accuracy of 83%. Although the accuracy of the 3D-CNN is 90%, the reduction in the complete two-step algorithm performance is from the SUDCS missing vessel segments, thus not passing them off to the 3D-CNN for perfusion analysis.

Our results indicate that the algorithm can be used for a reliable and reproducible analysis of microscopic images from intravital microscopic observations. Currently, the analysis of IVM video data can only be done by manually assessing the microvascular flow of blood in each vessel segment in the observed FOV. Although imaging the perfused vessels' geometric structure using the augmented SAD images helps to identify which vessels are actively perfused, each identified vessel segment needs to be evaluated separately. Since this involves a detailed visual inspection of the recorded videos, the analysis of these observations becomes an extended and time-consuming undertaking and can easily lead to analyzer fatigue and affect the performance and accuracy of the analysis^33–35^. Moreover, to improve the manual analysis accuracy, experiments are usually analyzed several times by multiple analyzers, which further compounds the laborious and time-consuming nature of this procedure.

We find that our algorithm reduces the analysis time of a typical 60-s experimental observation (involving up to approximately 2000 microscopic images) from 1–2 h to several minutes, thus significantly reducing the protracted character of the analysis procedure. Furthermore, it is essential to note that although the algorithm's overall accuracy is 83%, the algorithm cannot be affected by analyzer fatigue, and its performance and accuracy will be similar for each analysis cycle. Consequently, each observation in an experiment will be analyzed using the same parameters, allowing for effective comparison of the different observations during one experimental procedure. Once trained, the CNN will analyze all data without inaccurate analysis due to human fatigue and error.

Although the experiments in this study have been performed on the microvasculature in muscle tissue, the algorithm's principles are also applicable to other microvascular structure types and can be used for IVM investigations in other tissues. Therefore, future work involves further optimizing the complete algorithm, improving the pre- and post-processing algorithms, and further automatization to determine the optimal parameters for the SUDCS algorithm and test the algorithm on different tissues and imaging techniques.

## Methods

### Animal preparation

All experimental animal work described in this study was performed in accordance with the legal guidelines and regulations set by the Canadian Council of Animal Care (CCAC) and approved by the Animal Care and Use Committee (ACUC) of Western University, London, Ontario, Canada. All animal work in the study was carried out in compliance with the ARRIVE guidelines.

Specific Pathogen Free (SPF) male Sprague–Dawley rats, purchased from Charles River (Wilmington, MA, USA), were housed under standard conditions, i.e., water and food ad libitum, 12/12 light–dark cycle at room temperature. The animals were allowed to acclimatize for at least 72 h after arrival at our animal facility before entering the experimental procedures.

All animals (200–250 g body weight) were fully anesthetized with an isoflurane oxygen mixture (induction 4% isoflurane, maintenance 2% isoflurane) and breathing freely during the experiment. Body temperature was maintained at 37˚C using a rectal probe and an infrared lamp connected to an automated temperature monitor (TCAT-2 Temperature Controller, Physitemp Instruments Clifton NJ). A 24G intravascular catheter was placed in the tail artery connected to a pressure transducer to monitor blood pressure and heart rate using a rodent blood pressure analyzer (DMSI-400, Mircro-Med Inc, Louisville KY USA), as well as for fluid resuscitation and to facilitate taking small blood samples for blood gas analysis; a second 24G intravascular catheter was placed in the tail vein for fluid resuscitation and to enable the IV-injection of drugs if needed. Fluid resuscitation with heparinized (1U/ml) 0.9% sodium chloride through both catheters was used (arterial catheter 350 µl/100 g body weight/hr; venular catheter 250 µl/100 g body weight/hr), only to ensure patency of both the arterial and venular catheter during the experiment.

### Surgery

Microvascular observations are performed on EDL muscle as previously described^36^. Similar to earlier described, the EDL muscle is exposed and separated from the surrounding tissue, after which the distal tendon is tied and detached from the bone. After exposing the muscle tissue, the animal was transferred to an inverted optical microscope, gently positioning the exposed muscle on a thin glass coverslip. The exposed muscle was regularly super-fused with physiological saline and covered with saran wrap and a glass coverslip to isolate the muscle from the surrounding environment and prevent desiccation of the tissue.

**Figure 3:**
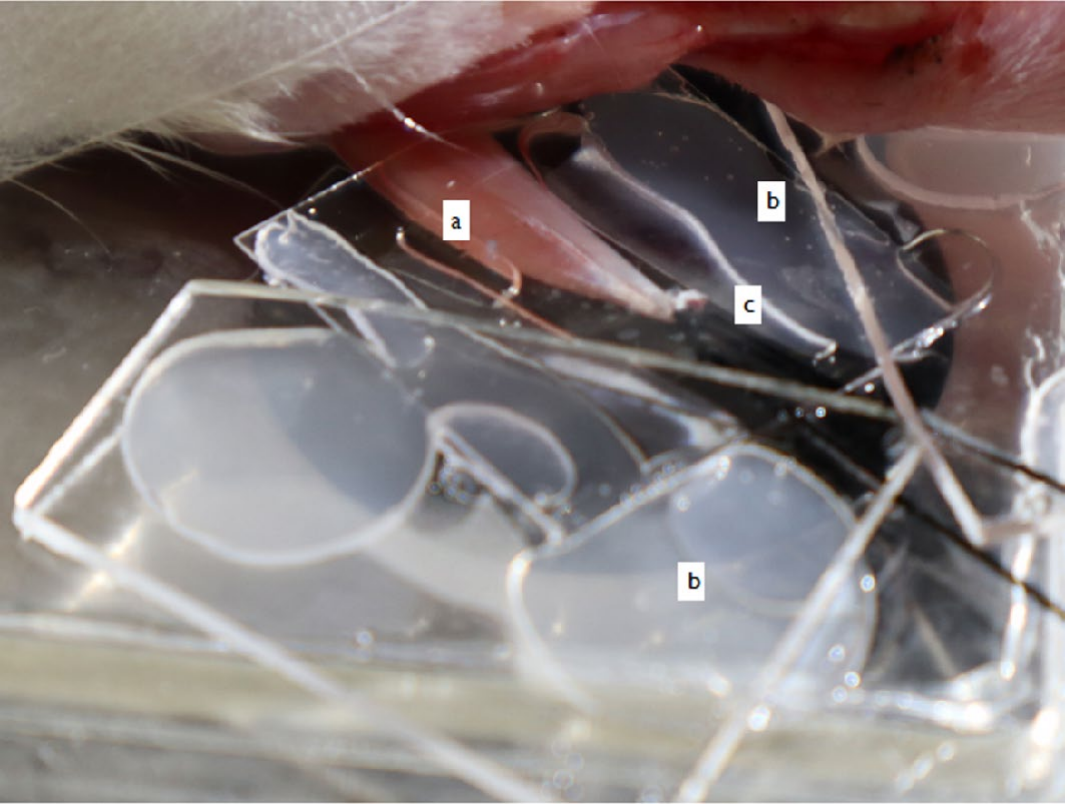
Tissue preparation for IVM. Exteriorized EDL muscle **(a)** prepared for intravital microscopy. In these observations. The muscle is covered with saran wrap to prevent desiccation and covered with a cover glass slip. Small drops of silicone sealant **(b)** are used to keep the glass coverslips in place. A suture **(c)** attached to the muscle–tendon is used to hold the muscle in place and keep the muscle at its approximately in situ lengths (image produced by author B. Janssen).

Tissue movement is minimized by using a suture at the tendon to hold the muscle in place and keep the muscle at its approximately in situ length without impeding microvascular blood flow (see Fig. 3).

### Brightfield microscopy

Observations of 60 s at 32.5 frames per second are made using an inverted microscope (Nikon Eclipse-Ti, Nikon Instruments, Melville, New York, USA), using an intravital microscopy adapted microscope stage. The EDL tissue is transilluminated using a 100 W xenon light source (PTI LPS 220, Horiba Scientific, Piscataway NJ, USA), combined with an optical light guide (Thorlabs, Newton, NJ, USA). The tissue is transilluminated with light in the range of 400–550 nm, while an additional filter (450 nm/20 nm band-pass filter; 450BP20; Omega Optical, Brattleboro, VT, USA) is placed in the light path directly in front of the camera. This approach enhances RBC visualization and prevents any tissue damage related to the UV and IR light emanating from the xenon light source. Intravital microscopic images (1000 × 1000 pixels; 16 bit) of the EDL microcirculation are acquired using a newly developed multispectral multi-camera imaging system (MSMC-23-1-A, Spectral Devices Inc., London, Canada) containing integrated 4 CMOS cameras (IMX249 sensor, Sony), and subsequently stored on a hard drive for later off-line analysis.

### Initial manual acquisition training data set

**Figure 4:**
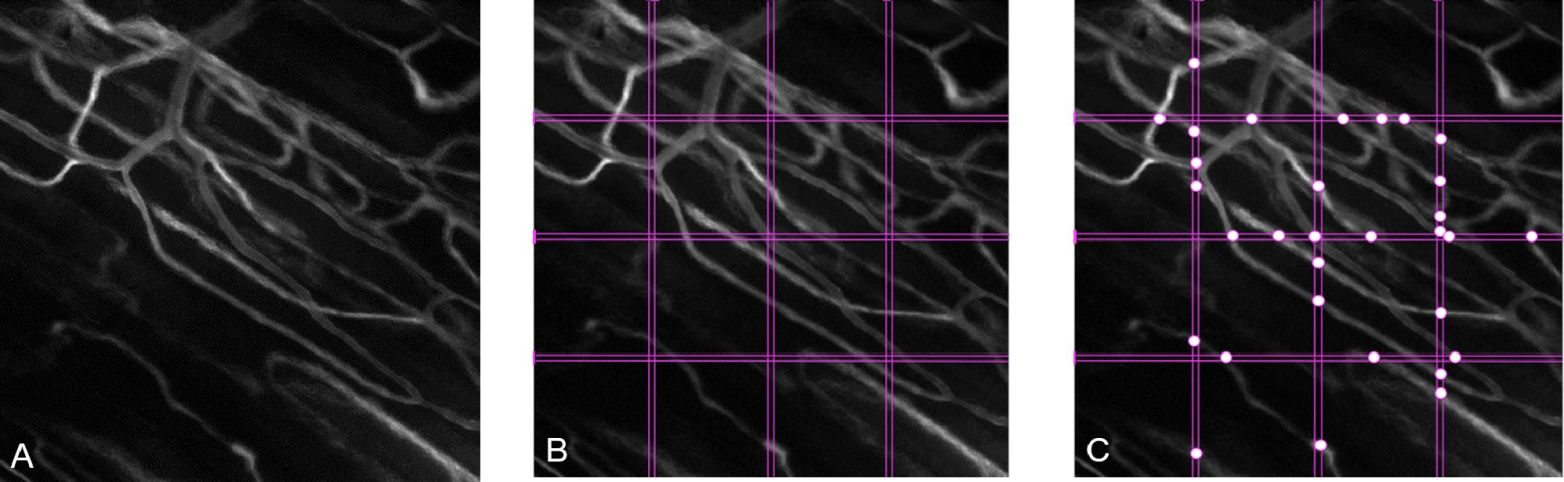
Image of the Sum of Absolute Differences. **(A)** Original SAD image (FOV: 400 × 400 µm). **(B)** Three sets of two equidistant horizontal and vertical lines acting as a visual reference. **(C)** Intersections of perfused vessels are manually identified between the lines and cross-referenced with associated intravital microscopic video recording^45,46^.

All captured images are pre-processed using in-house written Matlab-based software (Matlab 2020a, the Matworks Inc, Natrick, MA USA; https://www.mathworks.com), partially based on algorithms as earlier described^2^. It enables identifying vessel segments that are actively perfused by registering the absolute value of light intensity changes in the associated pixels due to flowing RBC movement. Adding up these values creates a Sum of Absolute Differences (SAD) images, allowing to visualize actively perfused microvessels (Fig. 4A).

The training data set used for algorithm training was acquired by manually analyzing the initially pre-processed IVM images from previous intravital microscopic observations of the EDL and using a previously described method^17^. In short, the SAD images were used to manually identify the perfused vessels in the Field of View (FOV) (Fig. 4A). Three equidistant horizontal and vertical lines acting as a visual reference to mark the spots where these lines intersect with perfused microvessels were superimposed on the SAD image (see Fig. 4B). To minimize the possibility of any misidentification resulting from a possible optical artifact, each identified perfused vessel segment in the SAD image is visually verified by cross-reference with the same vessel segment in the associated intravital microscopic video recording. Once verified, these intersections' location is then digitally recorded and compiled into a training set to train the machine-learning algorithm (Fig. 4C). IVM videos from either set were only used in the training or test dataset.

The final manually acquired dataset contained 32,213 labeled vessels generated from 458 IVM videos. The data set was then split by video to a training dataset of 392 IVM videos used to train the 3D-CNN algorithm. A separate test set containing 66 IVM videos was used to test the algorithm's performance.

### Two-Step algorithm

Manual analysis of the microcirculation in the IVM video incorporates two significant discrete analysis steps, i.e., identifying the layout of the vessel structures across in the FOV and determine whether these structures carry blood flow or are optical artifacts. The proposed two-step algorithm is designed using in-house written Matlab based software (Matlab 2020a, the Matworks Inc, Natrick, MA USA; https://www.mathworks.com) and mimics the manual analysis by initially using the SUDCS to detect vessel-structure, and layout, followed by applying a trained convolutional neural network to determine if the identified vessel structures contain blood flow or not.

### Image processing for the automated Two-Step analysis of perfused vessel segments

**Figure 5:**
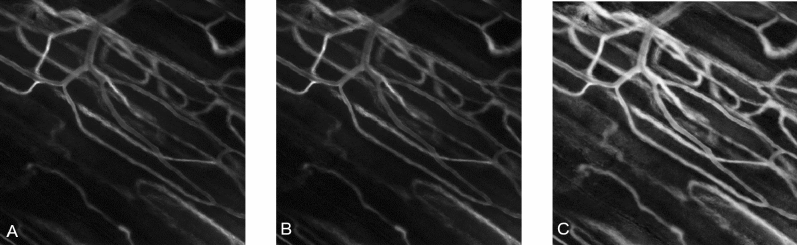
Image processing of the SAD image. **(A)** The SAD image of a FOV (400 × 400 µm) in the EDL muscle of a rat; visible are the highlighted structures associated with the vasculature. **(B)** Displays the SAD image after removing moving frames from the microcirculation video. **(C)** Presents the augmented SAD image after the pre-processing step with CLAHE is applied^45,46^.

The image data used to create the SAD image (Fig. 5A) is further processed to reduce any motion artifacts using a traditional block matching algorithm similar to the one used by^37^. It estimates the motion artifact in the image frames based on the first frame. It then removes any frame with a motion velocity greater than 0.3 pixels per second from the sequence of microcirculation images (Fig. 5B). Subsequently, the stabilized SAD image is further augmented by a Contrast Limited Adaptive Histogram Equalization (CLAHE), enhancing the highlighted vessel structures and improving their visibility (Fig. 5C). It facilitates the detection of vessel structures in the segmentation step of the two-step algorithm.

### Step 1: vessel segmentation; the steger unbiased curvilinear detector structures (SUDCS)

**Figure 6:**
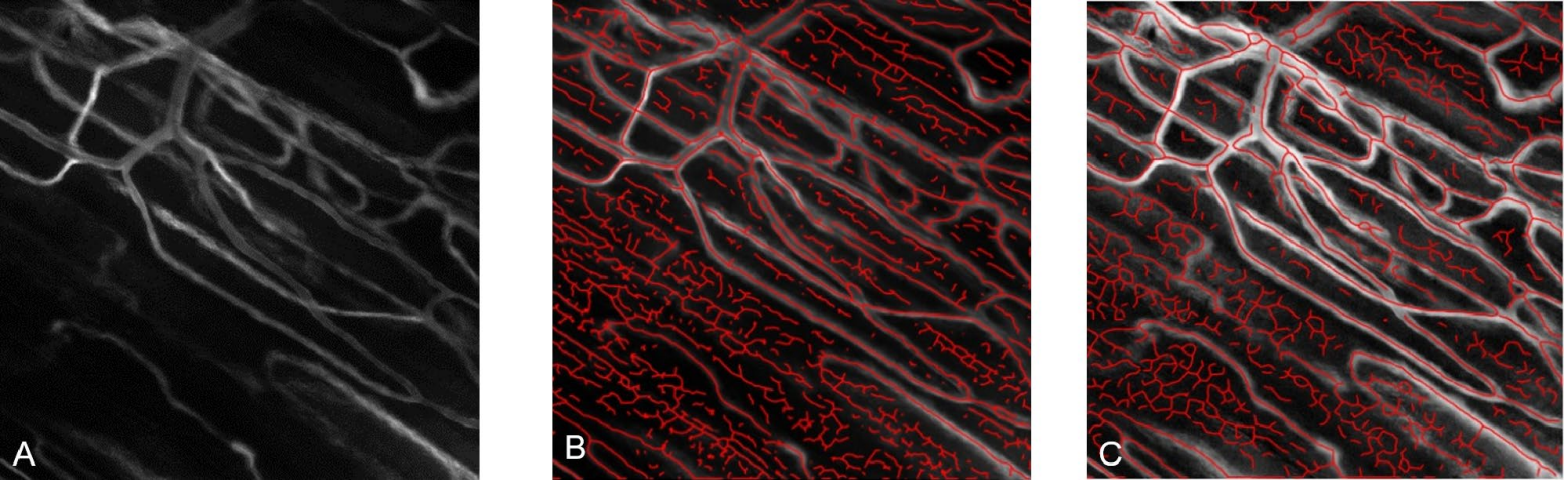
Overlay SAD and SUDCS. **(A)** Original SAD image (FOV: 400 × 400 µm). **(B)** An original SAD image with overlaid SUDCS vessel structure results (red lines). **(C)** Augmented SAD image after removing motion artifacts and CLAHE filtering overlaid with the modified SUDCS, a binary mapping (red line) is superimposed on the vessel structure. Note the false-positive segment structures, which will be removed in the 2nd step of the 3D-CNN^45,46^.

In this step, vessel-like structures are extracted from the processed SAD image by applying the SUDCS algorithm. This algorithm identifies the pixels in the image that exhibit vessel-like characteristics by examining the degree and direction of intensity changes^19^ in the image that was previously used to extract the vessel geometry of the retina^38^, resulting in a segmented vessel structure which comprises of both flowing vessels and non-flowing artifacts (Fig. 6A).

The identified structures are subsequently mapped into a binary image in which the value 1 represents a vessel structure, and a 0 represents a lack of vessel structure (Fig. 6B). To further improve the Steger Unbiased Curvilinear Detector Structures (SUDCS) algorithm, we included two additional modifying steps to the vessel segmentation algorithm. First, any discontinuities in the initial superimposed vessel segmentation are filled in the binary vessel image using a morphological-close operator^39^, followed by removing all segment structures shorter than 4 pixels (Fig. 6C) the results of the modified SUDCS superimposed on the augmented SAD image. Although the SUDCS algorithm's result contains false positive segment structures, it encompasses almost all correct vessel structures; the convolutional neural network for flow analysis (step 2 of the algorithm) will remove these incorrectly identified structures.

### Step 2: convolutional neural network for flow analysis

To differentiate between flowing vessels and artifacts in the vessel segmentation, a 3D-CNN was designed and trained with the training dataset obtained with manual analysis. The 3D-CNN's performance was assessed by comparison to a simple Logistic Regression model, in which each pixel value was represented as an input feature. This comparison is usually made to show the utility of the CNN over simpler machine learning technique^40^.

### Design of the spatial–temporal 3D convolutional neural network

**Figure 7:**
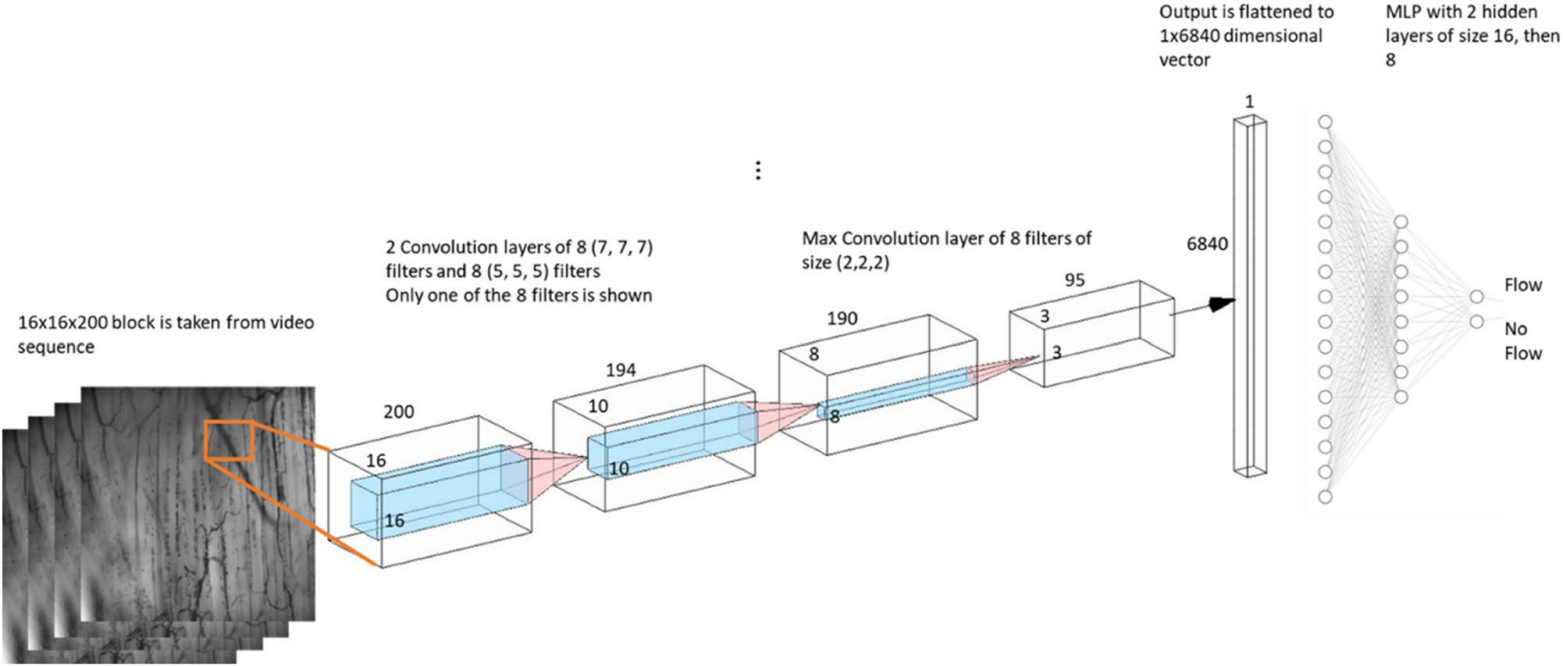
Visual representation of 3D-CNN architecture. The data is processed through a series of convolution layers, ultimately resulting in a flow or no-flow status in a particular vessel segment (image produced by author O. Mahmoud)*.*

The 3D-CNN architecture was designed to process data blocks generated from image data, consisting of a series of convolutional and pooling layers. Various windows of different sizes are convolved with the input data to create a compact representation (Fig. 7). Eventually, the layers' output is then "flattened" to a one-dimensional vector and passed into a connected multilayer perceptron, which will generate the final output after a series of learned weight multiplications.

Data blocks are created by defining a surrounding area of 16 × 16 pixels around each pixel on the identified intersections (Fig. 4B) between the equidistant lines and the vascular morphology (Fig. 6B) for 200 consecutive images. These three-dimensional (3D) 16 × 16x200 data blocks, each consisting of 65,536 data points, form the input for the spatial–temporal 3D convolutional neural network (3D-CNN). A 3D-CNN is a powerful tool for object recognition and has great potential for its use in computer-vision-based research since it is capable of assessing movement^24^, as well as changes in complex biological vessel structures^41,42^, and is capable of learning characteristics of the recorded images^43^.

The 3D-CNN network was developed (see "Training and Development of the 3D-CNN"), consisting of two hidden 3D convolutional layers, one with eight 7 × 7 × 7 convolutional filters, subsequently followed by a filter consisting of eight 5 × 5 × 5 convolutional filters. These convolutional filters are then followed by a max-pooling layer of eight filters of size 2 × 2 × 2, which work to reduce the representation of the convolutional layers' output, resulting in more efficient processing of the initial data blocks. The activation function used in all the convolutional layers was a Rectified Linear Unit function (ReLU). The max-pooling layer's output is then flattened to a data vector of size 6840 × 1, which acts as a compact representation of the pixels data block. This data vector is transferred through a traditional Multi-layer Perceptron (MLP) using a soft-max activation function to reduce this representation to a single value between 0 and 1, indicating the probability of flow in a vessel segment. Applying a threshold of 0.5 will return a binary representation indicating flow or no flow.

**Figure 8:**
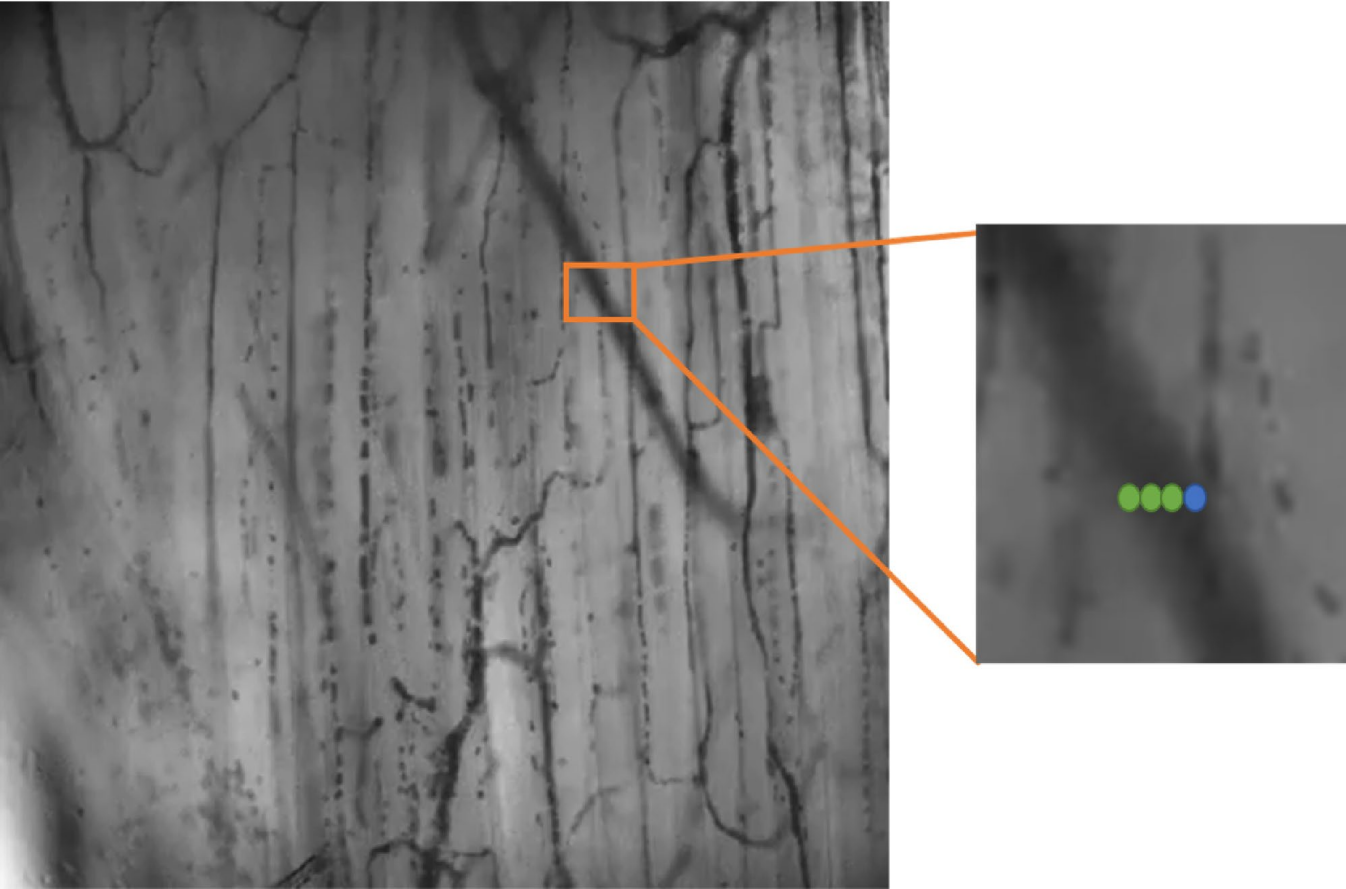
Pixel detection and 3D-CNN evaluation of a cross-section of a vessel segment. It shows a group of 4 pixels across a vessel, of which 75% (3 green pixels) are labeled as flowing, and 25% (1 blue pixel) is labeled as non-flowing. Since the threshold is set at 50%, this vessel is labeled by the algorithm as flowing^45,46^.

Moreover, as the 3D-CNN is applied to every pixel in a cross-sectional area of an identified vessel segment, a threshold was set that, if more than 50% of the cross-sectional pixels were classified as flowing, the vessel segment was identified as a segment with active blood flow (see Fig. 8).

### Training and development of the 3D-CNN

Training of the 3D-CNN was performed using the data labeled from the manual analysis of 392 IVM experimental videos. The 3D-CNN network was trained by minimizing the categorical cross-entropy and using it as the loss function. The Adadelta learning method was used as the training algorithm since it allows for an adaptive learning rate that requires no manual tuning of the learning rate^44^.

In our studies, the training step was done by subdividing the entire training data set of 392 experimental videos (27,809 data points) in batches of 30 data points. The accuracy of the 3D-CNN model was evaluated by computing the error rate after a full pass through the training data (1 epoch). This entire process was repeated for 100 epochs or until the filter weights of our model converged. The final accuracy of the 3D-CNN model was evaluated using the remaining 4404 data points. Assessment of the accuracy of the 3D-CNN after every epoch ensures that the training allows the 3D-CNN model to evolve and improve its's accuracy. The model architecture was chosen through tenfold cross-validation on the training set; the model showing the best performance was subsequently chosen for this study.

## Data Availability

All data generated and analyzed during this study are included in this published article. The image processing software for co-registration and removal of any tissue movement can be downloaded from the following links: https://github.com/OssamaMahmoud/2-Step-Microcirculation-Vessel-Detector^45^ and https://www.mathworks.com/matlabcentral/fileexchange/75197-2-step-microcirculation-vessel-detector^46^.

## Abbreviations

CLAHE: Contrast limited adaptive histogram equalization
CNN: Convolutional neural network
EPIA: Epipolar-plane image analysis
FOV: Field of view
FPR: False positive rate
FNR: False negative rate
IVM: Intravital video microscopy
MD: Microvascular dysfunction
MLP: Multi-layer perceptron
RBC: Red blood cells
SAD: Sum of absolute differences
SPF: Specific pathogen free
SUDCS: Steger unbiased curvilinear detector structures
TPR: True positive rate
TNR: True negative rate
tSICA: Temporal sidestream dark field image contrast analysis
